# Expert-based quantitative modeling for the probabilistic assessment of human factors risks related to physical literacy

**DOI:** 10.3389/fspor.2026.1818158

**Published:** 2026-05-11

**Authors:** Jiangping Fu, Bing Cao, Xiaojun Zhu, Yi Yu, Guanglin Bian, Zhi Sun

**Affiliations:** 1 Nantong Institute of Technology, Nantong, China; 2 Zhengde Polytechnic, Nanjing, China; 3 Jiangsu Open University, Nanjing, China

**Keywords:** cultural risks, human factors, physical activity, physical literacy, risk probabilistic modeling

## Abstract

**Background:**

The introduction of the concept of physical literacy (PL) has led to progress in the SDG3 goal area of human physical activity (PA). Safety culture theory recognizes PL indicators as human factors (HF) of PA and that there is a phenomenon of cultural risk. HF may pose potential risks to the implementation/effectiveness of PA, and there is an urgent need to explore risk mechanisms of HF in PL.

**Methods:**

The Perceived PL Instrument (PPLI) was chosen as the instrument to assess the measurement of HF in PA. A sample of 62 physical education/policy specialists was drawn using a simple sampling method. This study introduced the Decision-Making Testing and Evaluation Laboratory (DMTAEL) from a safety culture perspective and constructed the HF Risk Probability Assessment Model (HFRPAM) based on the PPLI's all-cause structure (two stages and six attributes) to quantitatively analyze the risk probability of HF. It then carried out the diagnosis (risk level), identification (risk weights and sensitivity), and optimization (paths) of HF risk probabilities.

**Results:**

The findings of the study observed that HFRPAM, as a perceived PL result of HF of PA, may be characterized by a risk probability. HFRPAM does not have a high-risk level. The risk level for the all-cause structure of HFRPAM was high correlation (≥4) and medium risk level 0.4803 (0.40 < *p* < 0.8). Results of the risk levels for high-correlation (≥4) of the secondary and tertiary indicators in the all-causal structure of HFRPAM: CS(X1) core stage has a high-risk level of 0.9838 (0.81 ≤ *p* ≤ 1.0), which is the highest. CS(X1) core stage is the single HF with the highest risk weight (12.82%). ES03(X8) knowledge and understanding was the HF with the highest sensitivity value/activity (0.3881) and the highest optimization index (0.5106).

**Conclusion:**

The results indicate that, as an exploratory study, the quantitative HFRPAM calculation method proposed in this study can serve as a knowledge translation for current structural models of perceived PA, providing a beneficial behavioral framework to complement the scientific validity of HF in the context of PA.

## 1 Introduction

Over the past 30 years, the introduction of the concept of physical literacy (PL) (1, 2) has brought about a new discourse on physical activity (PA) (3, 4). PL has become a topic and factor in the growing number of PAs in Physical Education (PE) (5). However, there is a distinct lack of understanding of PL and a lack of complexity theory and safety culture theory to guide the effect of Sustainable Development Goal 3 (SGD3) (6).

The introduction of PL has caused improvements in public health policy and public service policy (PE) for PA in many countries (7–11). PL is proposed to improve sedentary behavior and lack of PA globally to provide strong PA support for public health policy (12). PL also brings PA well-being to the physical health of the individual, targeting obesity and chronic diseases (cardiovascular disease) (13). In China's strategy for a strong sporting nation (the Healthy China 2030 Plan), PL indicators were proposed to help guide PA and achieve SDG3 (14).

PA policy changes or implementations have multiple human factor (HF) safety culture pitfalls (15). HF forward-looking or leading-edge goals/initiatives maybe can help organizational leaders further improve PA implementation of policies (16). The HF controversy over the philosophical properties of PL has been ongoing since its inception (17). HF measurement of PL, focusing on what is possible and what is not (6), and its effectiveness (10), has attracted the attention of safety culture scholars. From a safety culture perspective, there is still a controversy over the risk probability (RP) of HF between PL and PE, healthy PE (comprehensive PE), and high-quality PE (3–5, 9). PL has a metaphorical basis in PA (18), influenced by socio-cultural approaches/RP and holistic discourse/ HF (19). Under the HF influence of critical thinking in Australia and the UK (20–22), PL's inclusive ideology may be misguided by RP (23). Cultural labeling of PL (24), including various localizations of HF (25–27), is a phenomenon of safety culture, which also has RP characteristics. A meta-analysis of PE teachers showed PA measures of PL (structural modeling), which are worrisome (28). PE teachers are responsible for implementing national education policies and national public health policies at the grassroots level. The scientific validity (rationality) of the structural model of PL for PE teachers becomes an important factor in the sustainability of organizational/national public health/service policies, triggering safety culture concerns about the perception of PA (RP of HF).

Whitehead's 2-stage 6-attribute of perceived PL, as an effective way of assessing the cognitive structure of PA, have not been fully validated or observed (24–27). In a different and more straightforward expression (research gap/gap) is that HF interrelationships between Whitehead PL attributes are often hypothesized, but rarely tested for their safety culture characteristics through RP empirical methods. Describe more specifically the research gap/gap that this study intends to fill: The 2-stage 6-attribute of PL as a human factor outcome; This outcome has a RP characteristics; This characteristics include: risk level, risk weight, sensitivity value, and optimization index; This study exploratory analyzed, the 2-stage 6-attribute of PL as a RP characteristic of a human factor; High human factor (high relevance) does whether it brings about a high RP characteristics (high risk level, high risk weight, high sensitivity value and high optimization index). Based on this, the research objective of this study is to validate the quantitative relationship of HF's RP between the 2-stage 6-attribute of Whitehead's perceptual PL, and to validate the reliability of the cognitive results on PA from a safety culture perspective. In 2025 the study on SDG 3 intangible cultural heritage sports, conducted within the Decision-Making Trial and Evaluation Laboratory (DMTAEL), determined that a lack of PL may constitute a sustainable HF and possesses RP attributes (28). This study treats individual or collective perceived PL behavior as an intervention outcome of HF for PA with RP characteristics and is a SDG3 sub-influence with pragmatic value. Based on this, this study incorporates the DMTAEL from the value of practice (safety culture) perspective in sports medicine to observe the characteristics of safety culture in HF, including diagnosis, identification, and optimization of RP, on six attributes of PL in Whitehead.

**Figure 1:**
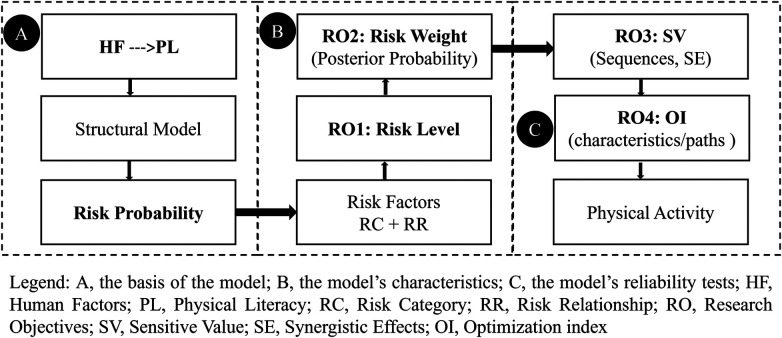
The evidenced logic of structural (human) factors of structural models.

Based on the above considerations, the research focuses on using the perceived PPLI structural model of PE teachers as a sample to construct the HF RP Assessment Model (HFRPAM). See Figure 1. This HFRPAM is used to quantitatively analyze the probability of HF risks, followed by the diagnosis (risk level), identification (risk weights and sensitivity), and optimization (paths) of these risks.

Based on the topic of the study, this study makes the following research objectives: (1) Risk Level: HFRPAM, as a perceived outcome of HF in PA, may be characterized by a risk level and may be assessed for risk level; (2) Risk Weights: the HF structure of the HFRPAM is likely to be characterized by risk weights for RP and can be quantitatively calculated; (3) Sensitive Values: the HF structure of HFRPAM may be characterized by sensitive values of RP and the activity of HF can be determined by the calculation of the sensitive values (sequences, simultaneous synergistic effects of multiple factors); (4) Optimization index: the HF structure of the HFRPAM may have optimizable characteristics/paths and can be identified by an optimization index.

## 2 Methods

### 2.1 Study design

This study employed an expert-based quantitative modeling approach, positing that HF can enhance the perception of PL and promote PA. It treated HF as a risk factor and further developed the HFRPAM to assess the mathematical relationship of RP for HF risk. Prior to the start of the study, this research was approved by the Zhengde Polytechnic Ethics Committee (ZDPC-2024PE-074).

There is a lack of quantitative research on PL. In order to better guarantee the scientific validity and rigor of the study, a detailed research design was developed, which primarily involved establishing an expert research team, designing the research procedures, and defining the research content in a step-by-step manner.

#### 2.1.1 Expert study group

This study established an expert working group to assist researchers in jointly ensuring the quality of the research, thereby enhancing and maintaining the reliability and validity of the overall research process (29).

In addition to all of the authors of this article, this research team also includes a professor of sports history, a safety expert, and a professor of PE (sports training). The research team's work consists of two parts. The first part involves participating in discussions on research design. The second part involves helping to design (adapt) questionnaires and distributing them in a targeted manner (specifying the type of research respondents, and making recommendations).

#### 2.1.2 Research procedures, steps, and content

The research team jointly determined the procedures, steps (sub-procedures), and content of the study design.

The research team jointly determined the procedures and content of the research design. The research design process is strictly arranged according to the logic of scientific research, and is divided into three stages: problem identification, problem analysis, and problem solving.

The first stage is the problem identification stage. During the problem identification stage, the primary tasks involved refining the research objectives, assigning roles and responsibilities, and defining objectives, all of which were accomplished through two steps.

The first step of the first stage involved establishing the personnel and objective procedures. The research team worked in an efficient and random network manner, with the corresponding author as the supervisor, and all authors participating equally in the work, each contacting experts within the research team. The research team jointly established a procedure for defining research objectives, with the following sequence: Research Objective 1 (Risk Level) → Research Objective 2 (Risk Weight) → Research Objective 3 (Sensitivity Value) → Research Objective 4 (Optimization Index).

The second step of the first stage involved refining the content framework of the research objectives.

The research team recognizes that the RP assessment of PL's structural model is based on the logic of whether PL is effective or not; put another way, PL's structural model may or may not be effective for PA implementation. Based on current PL research data, most studies have focused on cross-sectional observations, including groups of children, adolescents, and adults (25–27, 30). This study did not collect any useful data on whether sequence studies had been conducted. It is worthwhile to express further clearly that it is very crucial whether the test results of PL (structural modeling) are positively or negatively proportional to the implementation of PA. This is also the practical value of this research.

Based on the collected data, there seems to be an indirect relationship between the PL test results (structural model) and policy implementation, need to improve awareness of RP (19, 21, 22). More specifically, this refers to the potential benefits of PA resulting from the diagnosis, identification, and optimization of RP within structural models; or, in other words, it reflects how incorporating HF enhances people's understanding of PL. The research team unanimously agreed that this study was founded on the fact that the HF structure of PL facilitates the implementation of PA (there is a positive relationship between the two). A more nuanced and clearer description is that the structural model of PL (a feature of HF's RP) is scientifically sound (low risk) and will lead to good results in PA. In conjunction with the cognitive-behavioral theory, HF is a cognitive factor on PL (they are correlated), RP is an influence on behavior (PA), and the characterization of RP of HF is a materialization of the good effect of PL on PA.

For greater clarity, the PL structural model is called HFRPAM based on the safety culture viewpoint.

The third step of the first stage involved determining the structure of HFRPAM. Based on the refinement of the four research objectives and building on the work completed in the second step of the first stage, the research team collectively determined the structure of HFRPAM and clarified the correspondence between the structure and the research objectives. See Figure 1. The first part of the HFRPAM structure (Figure 1A) illustrates the basis of the model, which comprises three elements: HF, PL, and RP. The second part of the HFRPAM structure (Figure 1B) illustrates the model's characteristics, including the categories and relationships of risk factors, as well as the risk levels (Objective 1) and risk weights (Objective 2). The third part of the HFRPAM structure (Figure 1C) presents the model's reliability tests, including sensitivity values for RP (Objective 3) and optimization indexes (Objective 4), to illustrate the model's robustness results.

The second stage is the problem analysis stage. In the analysis problem phase, the main task is to quantify the characteristics of the RP of the HFRPAM, including the categories, relations, classes, probabilities (sequences/sensitivities) of the HF, and optimization (15, 16, 31). These works are divided into two steps.

The first step of the second stage involved defining the categories, relationships, and levels of HF, and configuring these as the categories, relationships, and levels of the variables.

Based on DMTAEL, the researchers created Figure 1, discussed it in depth with all the experts, and obtained their approval. The structural model of PL is the result of measuring tool verification, among which the Perceived PL Instrument (PPLI) is a leader (8, 32).The structural model of PPLI was designed and named based on Whitehead's concept of PL (26, 30, 32). Whitehead believes that PL encompasses the characteristics of individuals in holistic and lifelong physical activities, divided into the core stage (with three attributes: motivation, physical ability, and interaction with the environment) and the external stage (with three attributes: self-awareness, self-expression and communication with others, and knowledge and understanding), and emphasizes that the two stages are interrelated and dynamic (6, 24, 33–35).

The research team discussed and made an implicit logical decision to use the structural model of PPLI as the basis for the category and RP relationships of the HFs of HFRPAM, specifically labeled (coded) as two primary and six secondary HFs. See Table 1. The core stage is the first HF X1, and the external stage is the fifth HF X5.

**Table 1:** Table of human factors for structural models.

Primary human factors		Secondary human factors		Items
Structural factors	Encoding	Structural factors	Encoding	
Core stage	X1(CS)			
		Motivation	X2(CS01)	PL09c, 15c, 18b
		Confidence and physical competence	X3(CS02)	PL01c, 06c, 14d
		Interaction with the environment	X4(CS03)	PL03, 10c, 16d
External stage	X5(ES)			
		Sense of self and self-confidence	X6(ES01)	PL02a, 07a, 08a
		Self-expression and communication with others	X7(ES02)	PL11a, 12a, 13a
		Knowledge and understanding	X8(ES03)	PL04a, 05a, 17a

PL, physical literacy; (a) Traditional Chinese Physical Education Teacher Version; (b) Simplified Chinese Undergraduate Version; (c) Simplified Chinese Coach Version; (d) Simplified Chinese Faculty Version.

Based on the content of the first step of the second stage, this study included a total of 8 variables, comprising 2 first-level variables and 6 s-level variables. A more direct and detailed algorithm describing the relationships between the variables in HFRPAM, including the equations and the relationships between inputs and outputs, will be reported in detail in the data calculation section.

The second step of the second stage involved determining the probability of HF (sequence/sensitivities) and optimization, as well as defining the algorithm.

Using Bayesian network probability algorithms (BNPA) (36–38), the 5 × 5 matrix proposed by the International Organization for Standardization (ISO) 31,000 was incorporated as an RP risk standard to assess the level of HF (Research Objectives 1). It should be specifically noted that the rationale for this study's inclusion of ISO 31,000 (5 × 5 order matrix) as an RP risk criterion is that it represents a well-established analogous approach in the field of sport/ safety culture research (39).Through the algorithm of entropy reduction and mutual information (15), the RP of HF is evaluated, and the sequence of HF is ranked to obtain each risk weight (Research Objectives 2) and sensitivity value (Research Objectives 3). Through the comprehensive index algorithm (31, 40), the optimization (comprehensive) index of HF is evaluated to pro-pose an optimization path for HF (Research Objectives 4). Based on the results from the HFRPAM active sequence, an optimization path was established to explain robustness and demonstrate the model's reliability.

The third stage is the problem-solving stage. During the problem-solving phase, the primary focus is on reviewing the HF of the PL structural model, which includes discussing the benefits of implementing the PA and verifying the theoretical logic of the HFRPAM design.

The work in the third stage was completed in two steps.

The first step of the third stage: The problem-solving phase primarily involves reviewing the HF of the PL structural model. Using high-quality PE as a background (4), discuss the advantages (optimization of HF, Research Objectives 3, and Research Objectives 4) and limitations (risk level and weight of HF, Research Objectives 1, and Research Objectives 2) of the PL structural model for policy implementation.

**Figure 2:**
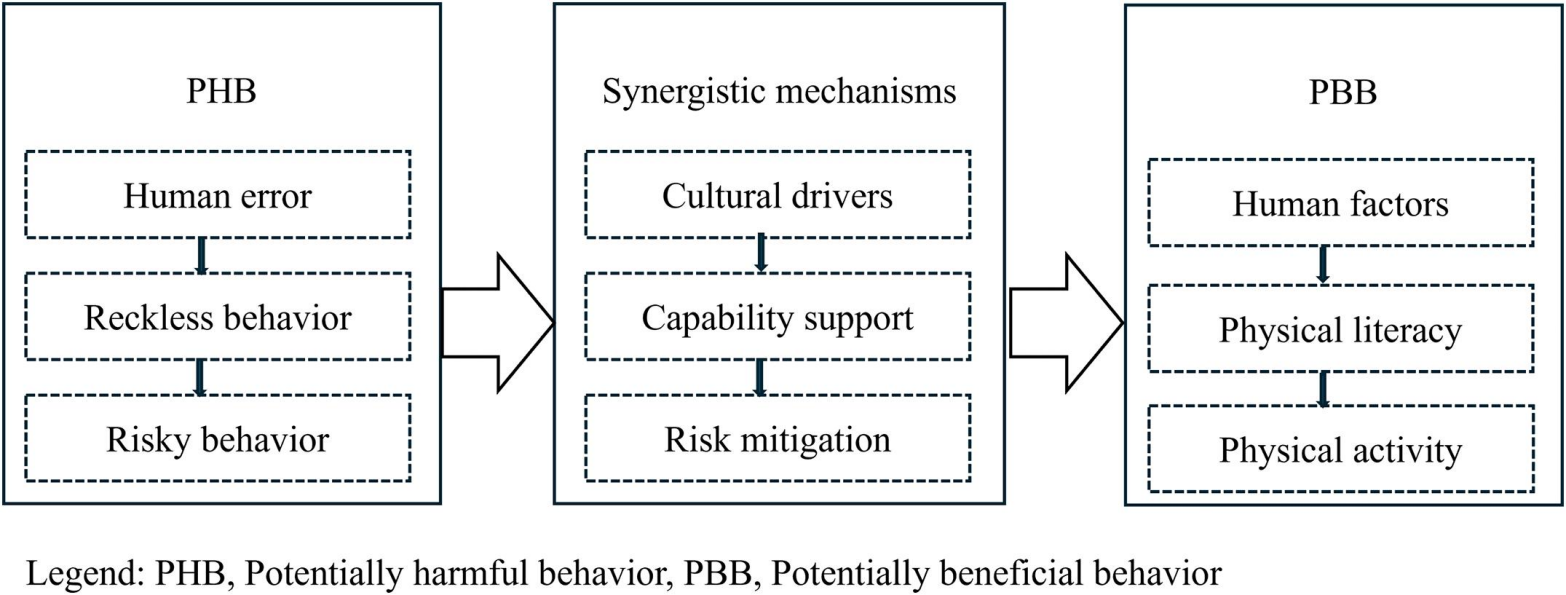
A three-dimensional synergistic mechanism framework/path for cultural security studies.

The second step of the third stage: The research team, together with all the authors, reviewed the theoretical rationale behind the HFRPAM design in this study. In the field of safety culture, the three-dimensional synergistic mechanism and path (Figure 2A)—centered on healthcare and patients and emphasizing practical application, which involves the sequence of cultural drivers → capability support → risk mitigation—may represent the most direct theoretical foundation for HFRPAM in this study (41). Taking the three-dimensional collaborative mechanism as a logical starting point, some researchers have proposed a framework and path for potential harmful behaviors within public health systems (Figure 2B), including human error → reckless behavior → risky behavior (31). The HFRPAM proposed in this study outlines a framework for potentially beneficial behaviors (Figure 2C), encompassing HF → PL → PA. Figure 2 further illustrated how the HFRPAM design in this study fills a gap in theoretical research [a framework for potentially beneficial behaviors, (31)], and demonstrated its value for practical applications [Compared with the sustainable development model of risk factors in intangible cultural heritage sports (28), this may represent the results of a longitudinal factor analysis of risk factors.], thereby completing the logical loop of the research design.

### 2.2 Research materials

PPLI is the direct material of this research. The structural model of PPLI is the indirect material (logical analysis material of HFRPAM) of this research.

#### 2.2.1 PPLI

In China, there are three versions of the PPLI: English (26), Cantonese (27), and Simplified Chinese (42). All three versions of the PPLI have undergone rigorous validity and reliability testing. The English version has Cronbach's alpha 0.73–0.76, the Cantonese version has Cronbach's alpha 0.51–0.82, and the simplified Chinese version has Cronbach's alpha 0.79–0.83. The three versions of the PPLI have different numbers of items, with the Cantonese version having 9 items and the other two versions having 18 items. To maintain the rigor of scientific research and in accordance with the study's objective (the full-factor structure of the PPLI structural model), this study selected the English version of Whitehead's 18 items.

The structural model of PPLI provide a comprehensive analysis of the structures observed in both the English and Simplified Chinese versions, serving as the basis for version selection and offering strong evidence for the validity of the all-cause structure of HFRPAM. Among them, the English version (PE Teacher) of the structural model consists of 3 dimensions and 9 items, claiming to have detected the core stage of Whitehead's PL concept, and naming it motivation, physical ability, and interaction with the environment. The simplified Chinese version (under-graduates) of the structural model is a three-dimensional, nine-item model that claims to have detected the outer core stage of Whitehead's PL concept, naming it self-awareness, self-expression and communication with others, and knowledge and understanding. The Simplified Chinese Coachman version of the structural model is a 4-dimensional, 9-entry model that claims to detect the phenomenon of the core and external stages of Whitehead's conception of PL intermingling. The Simplified Chinese faculty version of the structural model is a 4-dimensional, 12-entry model that claims to detect the phenomenon of the core and external stages of Whitehead's concept of PL intersecting, and is named after self-confidence, motivation, physical competence, and knowledge and understanding. This study included the PPLI structural model with 6 dimensions and 18 items as the standard for this study. For clarity, this study introduces Whitehead's PL concept, treating the core stage and the external stage as primary risk factors, with the corresponding six dimensions divided into secondary risk factors. In addition, the research objective of this study includes calculating the interrelationships between all HF, so continuous sequence coding (X1–X6, ensured that 18 entries were not duplicated) was performed (see Table 1).

#### 2.2.2 HFRPAM

Detailed mapping of the PPLI structural model to the HFRPAM structure: The primary HF was divided into X1(CS) and X5(ES), where X1(CS) comprises 3 secondary dimensions and 9 items [X2(CS01; PL09, 15, and 18), X2(CS02; PL01, 06, and 14), and X3 (CS03; PL03, 10, and 16)], while X5(ES) comprises 3 secondary dimensions with 9 items [X6(ES01; PL02, 07, and 08), X7(ES02; PL11, 12, and 13), and X8(ES03; PL04, 05, and 17)].

#### 2.2.3 Cross-cultural

The PPLI is a standard 5-point Likert scale (1 = strongly disagree, 5 = strongly agree). This study adapted PPLI and translated it from English to Chinese and backwards (bidirectional translation and mutual verification).

However, based on our research objectives, this study made four modifications (three settings and one adjustment) when creating the questionnaire.

The first setting is the introduction section of the questionnaire. In the introduction section of the questionnaire, the researchers first outlined the basic research requirements (ethical review and informed consent).

The second setting is the basic information section of the questionnaire. In the basic information section of the questionnaire, researchers set demographic parameters for the research sample (respondents), including age, gender, role, academic, professional service, and research direction. This study does not intend to examine sociological variables (demographic data). The basic information section has been added solely for the purpose of screening qualifications.

The third setting is the special instructions section of the questionnaire. In the introduction to the item, the researcher made a special note. Special note: Inform respondents to assess the risk of interrelationships between items from a crisis/safety perspective (1 = strongly disagree, 5 = strongly agree). To be more specific, each respondent (expert) was asked to rate the impact of HF (RP/cultural risk) on a 5-point Likert scale based on two primary variables and six secondary variables.

The fourth step of adaptation involves adjusting the dimensions and order of the items in the PPLI. List the core stage as the first item, followed by the three dimensions of the core stage, then the external stage, and finally the three dimensions of the external stage. Each dimension was presented with items, but no evaluation was required (see Table 1).

### 2.3 Research sample and data collection

#### 2.3.1 Research sample

**Figure 3:**
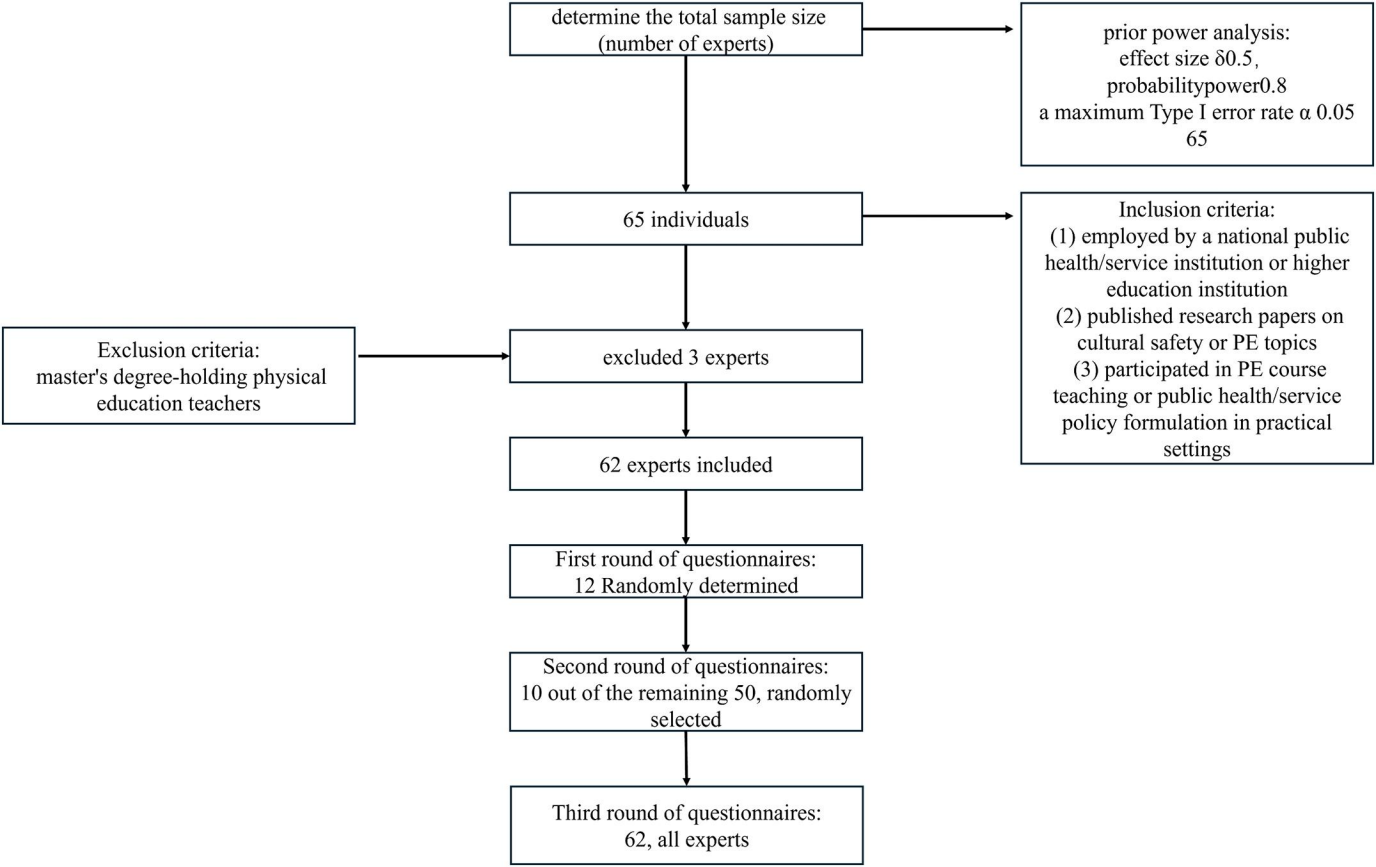
The participant-flow diagram.

This study sample comes from a group of industry experts (PL, cultural security, and PE, Figure 3). Regarding the selection of the study sample, based on a dual approach combining exploratory scientific research and expert consensus, this study employed a specific population/simple sampling method.

The corresponding author and the first three authors, as the sampling research team, completed the sampling work. The sampling research team consisted of two leaders from educational/PE organizations, one cultural safety researcher, and one PL researcher.

Corresponding authors are requested to ask members of the sampling research team to provide/establish a group of industry experts based on three criteria: (1) employed by a national public health/service institution or higher education institution, (2) published research papers on cultural safety or PE topics, and (3) participated in PE course teaching or public health/service policy formulation in practical settings.

The corresponding author asked two members of the sampling team to search six major Chinese and English databases—including Web of Science, Scopus, PubMed, IEEE Xplore, ScienceDirect, and CNKI (Chinese)—in accordance with the criteria for the expert panel, and to each provide a list of 100 experts. The corresponding author then cleaned the data from the list of 200 experts (200 = 100 + 100)—primarily by removing duplicate entries—and assigned computer-generated random codes to each expert. Finally, the list and its ranking were determined based on these codes.

The sampling team conducted a prior power analysis using Jamovi software version 2.6.26 to determine the total sample size (number of experts). This study included/set up an *F*-test and analysis of variance (repeated measures), within-subjects test, with effect size δ0.5, probability power 0.8, and a maximum Type I error rate α 0.05. Calculations indicated a total sample size of 51. This study further accounted for a 20% dropout rate (based on prevailing standards), necessitating an additional 11 experts (sample: 11 > 51 × 0.2). Consequently, the final total sample size was determined to be 62 (62 = 51 + 11).

The sampling research team provided a list of 65 individuals. Following discussions with the research team, it was unanimously agreed that 62 individuals would be included (3 master's degree-holding PE teachers were excluded).

Additional Note: The rationale for incorporating a prior power analysis into this study is based on the research team's comprehensive consideration of five factors, including: (1) the expert consensus method; (2) the 8EPP principle of medical models (eight events per candidate predictor; one model parameters—HF; assuming an incidence rate of 1, a sample size of 8 is required); (3) Identification of only high-confidence variable associations (the 8 all-cause risk weights in the PPLI structural model); (4) Use of exploratory Bayesian network modeling methods; and (5) No causal inference regarding the network structure of the structural model. Therefore, the sample size calculation based on prior power in medical research was incorporated.

#### 2.3.2 Data collection

The authors conducted the distribution and collection of questionnaires. The fourth author used the Wenjuanxing platform (tool) of the electronic QR code based on Table 1, the questionnaire was distributed and collected. Each respondent (industry expert) generates one data point after completing the questionnaire. The fourth author conducted three rounds of questionnaire distribution and collection, with an interval of two weeks between rounds. After collecting the raw data, the fourth author handed it over to the fifth and sixth authors (to prepare the data calculations).

Clear instructions, three rounds of questionnaires were set up for distribution and retrieval to achieve rigor in scientific research.

In the first round of the questionnaire, 12 respondents were randomly selected among 62 experts to validate the results of the cross-cultural study (English-Simplified Chinese translation). In the third part of the questionnaire, semi-open-ended items were set up, “please fill in the number of the entry that you think has unclear linguistic expression”, and the criterion for inclusion was that if any expert filled in any entry, the first round of the questionnaire would be redone for 7 consecutive days or until sufficient feedback has been collected to support decision-making.

For the second round of the questionnaire, 10 respondents were randomly selected from the remaining 50 (50 = 62–12) to validate the questionnaire's content validity index (CVI). In the third part of the questionnaire set up semi-open items, “please fill in the number of the entry that you think should be adjusted (e.g., PL01 belongs to entry 2)”, the criterion for inclusion is that if any expert fills in any entry, the second round of the questionnaire will be redone until all respondents agree.

For the third round of questionnaires, the final version of the questionnaire (cross-culturally validated and endorsing the categorization of items/items) was ad-ministered to all 62 experts in the third round.

### 2.4 Data calculation

The research team jointly determined the tools, steps, methods, indicators, and standards for data calculation. This study selected Jamovi (version 2.6.26) and Excel (version 2019) as the tool for data calculation (43).

#### 2.4.1 Data cleansing

**Figure 4:**
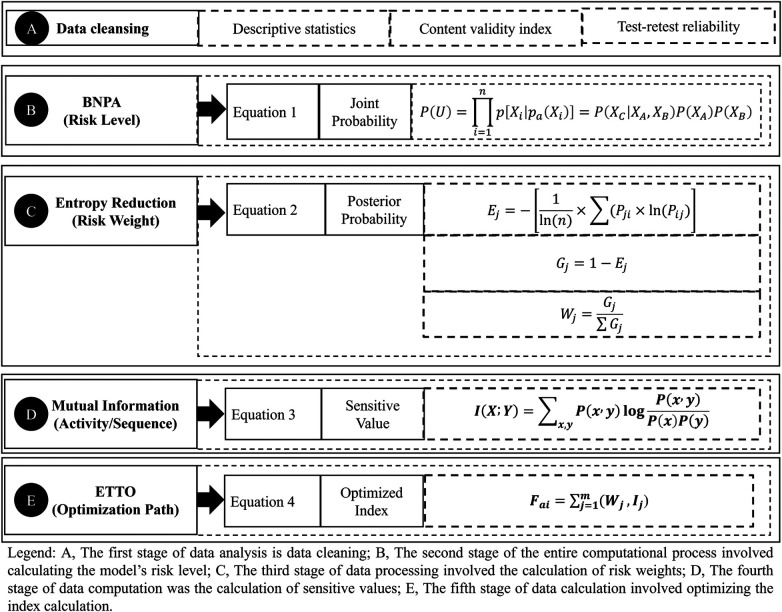
Methods, indicators, and standards for data calculation.

The first stage of data analysis is data cleaning (Figure 4A), which consists of four sub-steps: descriptive statistics, content validity index, test-retest reliability, and structural validity.

**Descriptive statistics:** Descriptive statistics are divided into two sections: cross-cultural validation and demographic information. **Cross-cultural validation:** The cross-cultural validation calculations involved calculating the response rates for the three rounds of questionnaires as percentages. **Demographic Information:** Demographic information calculated percentages of respondents' demographic variables, including age, gender, role, academic, professional service, and research direction.

**Content validity index:** The content validity of the questionnaire was assessed using the CVI, which was the item level (I-CVI ≥ 0.8, acceptable) and scale level (S-CVI ≥ 0.9, acceptable). The internal consistency of the questionnaire items was assessed using Cronbach's alpha index (>0.7, acceptable) (44). To ensure consistency in the model's sampling results across different chains and thereby verify the model's stability and reliability, the Rhat statistic (Gelman-Rubin diagnostic) was incorporated into the convergence check to measure the divergence between chains. A Rhat value close to 1 (<1.1) indicates that convergence has been achieved across the chains.

**Test-retest reliability:** The test-retest reliability of the questionnaire was calculated using the intraclass correlation coefficient (ICC, ≥0.5, acceptable), and the variability of all items was calculated (*p* < 0.001, acceptable) (44, 45).The data model (ICC) was tested for normality by incorporating the Kolmogorov–Smirnov test (sample size >50), absolute kurtosis (<10) and absolute skewness (<3), normally distributed (two-way, generally acceptable) (44).

**Structural Validity:** To validate the construct validity, Exploratory factor analysis and confirmatory factor analysis were performed separately for construct validation to further establish the construct validity of a new measurement instrument (44). Exploratory factor analysis incorporated Bartlett's test of sphericity (*p* ≤ 0.05), Kaiser-Meyer-Olkin index (KMO, >0.6), and factorizability of the correlation matrix (*r* ≧ 0.13) (44, 45). Confirmatory factor analysis using the maximum likelihood method, incorporated factor loading of all items (>0.4), root mean square error of approximation (RMSEA, <0.1), parsimonious normative fit index (PNFI, ≧0.5), comparative fit index (CFI, >0.95), relative chi-square (X2/df; <3), Standardized Root Mean Residual(SRMR, <0.01), Tucker–Lewis index(TLI, >0.95) (44, 45).

In the first stage of the calculations, the fifth and sixth authors were responsible for the initial data analysis. The fourth author reviewed the results, organized the data, and transferred it to facilitate the next phase of the calculations.

#### 2.4.2 Risk level

The second stage of the entire computational process involved calculating the model's risk level (Figure 4B), which was incorporated into the BNPA algorithm framework.

Using the Bayesian probability algorithm in Equation 1 (36, 37), the posterior probability of HF was calculated. The 5-point Likert-scale PPLI was incorporated as the criterion for the correlation between HF and perceived physical fitness to determine the HF risk level in the HFRPAM. The following categories were assigned: 1 (very low correlation), 2 (low correlation), 3 (medium correlation), 4 (high correlation), and 5 (very high correlation). Additionally, the correlation of the three anchor points for HF within the all-cause structure of the HFRPAM was assessed (low correlation, ≤2; medium correlation = 3; high correlation ≥ 4), and the 2-anchor correlation for secondary and tertiary HF (no correlation, ≤2; and positive correlation, ≥4).

Based on the 5 × 5 matrix framework proposed by the International Organization for Standardization (ISO) 31,000 (32), the equidistant quantification of HF risk levels is set at 0.2. Combined with the posterior probabilities of risk factors, the risk levels incorporated into the all-cause structure of the HFRPAM can be classified into a low-risk level (*p* ≤ 0.4), a medium-risk level (0.40 < *p* < 0.8), and a high-risk level (0.81 ≤ *p* ≤ 1.0); When incorporating Level 2 and Level 3 HF, the HF risk levels are defined as risky (*p* > 0.4) and non-risky (*p* ≤ 0.4).

A detailed indication of the entire BNPA designation and calculation details. This study incorporated EXCEL version 2019 as an aid to the calculations. After data col-lection was completed, it was sorted by columns (metrics) and rows (data) (state space and discretization rules), data cleaning was performed, metrics frequency (node set) was calculated, and nodes were included with low correlation (<3), medium correlation (=3), and high correlation (>3) using the COUNTIFS () formula. The logic of our joint probability calculation is based on the joint dataset of core stage metrics (X1) including X2, X3 and X4, and the joint dataset of external stage metrics (X5) including X6, X7 and X8, which are used as the antecedent nodes of the conditional probability (the logical basis of the conditional probability table), and the posterior probability of the HF are included as the result of the joint probability. Joint probability (posterior probability) is calculated by incorporating (prior probability * likelihood probability)/marginal probability. It is also stated that according to the information we have, it seems as if our team's study is the first thematic study to propose the phenomenon of RP of PL; so, there is no mature data table for the prior probability of PL. The *a priori* probability calculation incorporates the logic of calculating the joint probabilities, the ratios and nodes that incorporate the high, medium and low probabilities, using COUNTIFS (indicator dataset, “>3”)/COUNTA (frequency dataset of the joint dataset of the indicators). Likelihood probabilities were calculated by incorporating the ratios and nodes for the high, middle and low probabilities using COUNTIFS (indicator dataset, “>3”)/COUNTA (frequency dataset for the joint dataset of the 6 categories of indicators). Calculation of marginal probability incorporates (prior probability * likelihood probability) + [(1—prior probability) * inverse probability]. The calculation of inverse probability incorporates 1-likelihood probability. As a special note, BN's convergence check incorporates a Rhat (Gelman-Rubin diagnostic) that sets the causal structure (X1 → X2, X3, X4; X5 → X6, X7, X8) to measure the differences in the chains, with a Rhat value close to 1 (<1.1) indicating that convergence between the chains has been reached.

The calculations for the second stage and the verification of the results were performed by the corresponding author and the first author, respectively.

#### 2.4.3 Risk weight

The third stage of data processing involved the calculation of risk weights (Figure 4C), which was incorporated into the Entropy Reduction algorithm framework.

The RP (incidence rate) of HF is calculated using the entropy reduction Equation 2, and the algorithm of posterior probability (15), and the HF are ranked in order.

This is a detailed indication of the entire entropy reduction specified and calculated in detail.

This is a detailed indication of the entire entropy reduction specified and calculated in detail. Prior to the calculation, the BNPA dataset (EXCEL table), was processed by transposition, normalization [Eq. MMULT ()], normalization [Eq. MUNIT ()], and reversibility [Eq. MMULT ()], a total of six steps, in order to achieve the binning/discretization, bias correction for MI. In the process of entropy reduction, the first step calculates the a posteriori probability by incorporating the difference between the maximum and minimum values of the dataset as the binning intercept [Equation: Difference = MAX()-MIX()], and further calculates the probability of each value in the dataset (Equation: Probability = Value/Difference); in the second step, a transposition of the dataset is carried out into the probability of the dataset listed in columns of each variable; in the third step, the Calculate the lnpij portion of Equation 2 (formula: IF[cell value = 0, 0, cell value * LN(cell value)]; Step 4, Calculate the moderating coefficient [Equation: 1/LN()]; Step 5, Sum and calculate the entropy value, Ej (Equation 2); Step 5, Difference coefficient, gj = 1—ej; Step 6, calculate the weights, wj = gj/Σgj.

The calculations for the third stage and the verification of the results were performed by the corresponding author and the second author, respectively.

#### 2.4.4 Sensitivity values

The fourth stage of data computation was the calculation of sensitive values (Figure 4D), which was incorporated into the Mutual Information algorithm framework.

The sensitivity values of HF are calculated using the mutual information Equation 3 (15) to assess the activity (sequence) of HF. The higher the sensitivity value, the stronger the HF activity, which requires close attention/reinforcement/analysis.

This is a detailed indication of the entire mutual information specified and calculated in detail.

In the process of calculating mutual information, the first step is to use the Jamovi tool's packet in R language (infotheo), set the binning rules (full factors X1-X8, Equation 3), and quantify the conditional probability tables (CPTs) between the variables; in the second step, according to the CPTs, the sensitivity values are calculated, and based on the affiliation of the HF of the HFRPAM, X2-4 are the sub-indicators of X1 and X6–8 are sub-indicators of X5, which are included in the average value for calculation.

The calculations for the fourth stage and the verification of the results were performed by the corresponding author and the third author, respectively.

#### 2.4.5 Optimized index

The fifth stage of data calculation involved optimizing the index calculation (Figure 4E).

By incorporating the efficiency-thoroughness trade-off (ETTO) principle in safety management literature (45), this study determined the comprehensive index algorithm Equation 4 (31, 40), calculated the optimized (comprehensive) index of HF, and evaluated the optimized path of HF. The higher the optimization (comprehensive) index, the higher the value of the optimization path of the HF, and it should be selected as a priority.

The details of the entire ETTO/optimization index designation and calculation are shown in detail.

Based on mutual information calculation, in the first step, the designation of the ETTO/optimization index incorporates the sum of the sensitivity value and the risk weight as a criterion, using Equation 4, to calculate the optimization index; in the second step, according to the numerical magnitude of the optimization index, the ranking of the all-cause system (all risk factors/indicators) is carried out (the active sequence, the first to the eighth); and in the third step, based on the results of the active sequence, the optimization path is set (the interpretation of the robustness) and determine the hidden characteristics of HF based on the properties of HFRPAM.

The computational steps for the fifth stage were carried out by the corresponding author, after which the research team collaborated to analyze and interpret the results of the data calculations.

## 3 Results

### 3.1 Sample data

#### 3.1.1 Cross-cultural validation

In January 2025, a sample of the expert group was taken. The first round of questionnaire distribution was carried out to 12 respondents and 12 questionnaires were returned, with a recovery rate of 100%; after sorting and calculating, there were no items in the semi-open part of the questionnaire filled in by any of the questionnaires, which indicated that the results of the adapted questionnaire's English-Simplified Chinese cross-translation were recognized by the expert group for further research. A second round of questionnaires was distributed to 10 respondents, and 10 questionnaires were returned with a 100% recovery rate. A third round of questionnaires was distributed to 62 experts, and 62 were returned, a 100% recovery rate.

#### 3.1.2 Demographic information

The demographic information from the questionnaire was calculated, as shown in Table 2.

**Table 2:** Descriptive statistics of experts from questionnaire.

Items		*N*	%	Items		*N*	%
Gender	Female	37	59.68	Service	3 years or less	12	19.35
	Male	25	40.32		4–5 years	27	43.55
Ages	60 years and above	12	19.35		6 years (above)	23	37.10
	41–59 years	23	37.10	Vocational	Advanced	26	41.94
	31–40 years	24	38.71		Intermediate	27	43.55
	25–30 years	3	4.84		Beginner	9	14.52
Roles	policy experts	21	33.87	Research	Philosophy	17	27.42
	Education experts	21	33.87		Teaching (training)	18	29.03
	STE	20	32.26		Sports culture	16	25.81
Academic	Bachelor(below)	11	17.74		Safety education	11	17.74
	Master	28	45.16				
	Doctoral	23	37.10				

Remarks: Total *N* = 62; *N*, number of each item; %, percentage of each item; STE, sports training experts.

(1) Gender: Of the 62 respondents, 37 were female (59.68%) and 25 were male (40.32%). (2) Age: 12 people aged 60 and above (19.35%), 23 people aged 41–59 (37.10%), 24 people aged 31–40 (37.81%), and 3 people aged 25–30 (4.84%). (3) Roles: 21 policy experts (33.87%), 21 education experts (33.87%), and 20 sports training experts (32.26%). (4) Academic: 11 people with bachelor's degree or below (17.74%), 28 people with master's degree (45.16%), and 23 people with doctorate (37.10%). (5) Years of service: 12 people with 3 years or less (19.35%); 27 people with 4–5 years (43.55%); 22 people with 6 years or more (37.10%). (6) Vocational: 26 people at advanced level (41.19%), 27 people at intermediate level (43.55%), and 9 people at beginner level or below (14.52%). (7) Research directions: Sports philosophy (history) 17 people (27.42%), sports teaching (sports training) 18 people (29.03%), sports culture 16 people (25.81%), safety education 11 people (17.74%).

#### 3.1.3 Reliability test

The Cronbach's alpha coefficient of the questionnaire was 0.84 (>0.7), indicating that the internal consistency of the questionnaire items was stable and acceptable. The content validity results of the questionnaire were acceptable, with I-CVI of 0.83 (≧0.8) and S-CVI of 0.94 (≧0.9) (44).The retest reliability results of the questionnaire were acceptable, with ICC ranging from 0.63 to 0.84 (≧0.5) and all items were significant (*p* < 0.001) (44, 45).The distribution of normality (two-way) of the ICC model was generally acceptable, incorporating 496 (496 = 62*8) data for all 8 indicators, with a mean of 2.96, a standard deviation of 1 (45), and a Kolmogorov–Smirnov test of kurtosis with an ab-solute value of 1.33 (<10) and skewness with an absolute value of 0.07 (<3) (44).

#### 3.1.4 Internal consistency

Mean values of the 8 HF 1.79–3.90, with X7 (Self-expression and communication with others) 3.90 (highest, highest possible risk) and X6 (Self-awareness and self-efficacy) 1.79 (lowest, lowest possible risk); standard deviation 0.90–1.59 (44, 45).

The Rhat of the BN's convergence check (Gelman-Rubin Diagnostics) to measure chain differences, Rhat values (Point est. 1.024, Upper C.I. 1.071), close to 1 (<1.1) indicate that convergence has been reached between chains (44, 45).

#### 3.1.5 Structural validity

Principal component analysis of PPLI Structural Model was calculated by exploratory factor analysis and confirmatory factor analysis (see Table 3).

**Table 3:** Structural validity by exploratory factor analysis and confirmatory factor analysis.

(A) Exploratory Factor Analysis of subjective well-being									
Sign	F1	F2	F3	F4	F5	F6	C (h²)	CITC	SA
PL01	0.49	0.49	0.36	0.31	0.31	0.35	0.94	0.96	0.92
PL02	0.21	0.24	0.14	0.14	0.10	0.81	0.80	0.56	0.93
PL03	0.33	0.296	−0.05	0.31	0.69	0.03	0.78	0.61	0.93
PL04	0.44	0.627	0.24	−0.06	0.19	0.22	0.73	0.69	0.93
PL05	0.32	0.26	0.71	0.19	0.13	0.02	0.73	0.64	0.93
PL06	0.61	0.21	0.40	0.11	0.03	0.28	0.67	0.66	0.93
PL07	0.12	0.17	0.33	−0.03	0.78	0.15	0.78	0.53	0.93
PL08	0.42	0.22	0.39	0.43	0.28	−0.17	0.66	0.62	0.93
PL09	0.44	0.42	0.05	0.26	0.28	0.28	0.59	0.68	0.93
PL10	0.19	0.65	0.14	0.14	0.38	0.12	0.66	0.65	0.93
PL11	0.33	0.73	0.01	0.24	0.21	0.09	0.76	0.68	0.93
PL12	0.59	0.09	0.06	0.28	0.20	0.47	0.69	0.64	0.93
PL13	−0.01	0.37	0.23	0.63	0.02	0.37	0.72	0.56	0.93
PL14	0.14	−0.01	0.62	0.19	0.41	0.36	0.74	0.58	0.93
PL15	−0.02	0.63	0.47	0.26	−0.09	0.13	0.71	0.53	0.93
PL16	0.51	0.28	0.38	0.08	0.16	0.17	0.54	0.63	0.93
PL17	0.83	0.19	0.11	0.09	0.18	0.03	0.79	0.62	0.93
PL18	0.21	0.11	0.16	0.86	0.14	0.11	0.85	0.56	0.93
KMO	0.89								
BT	0.00								
C%	72.96								

(B) Confirmatory Factor Analysis						
Index	*χ*^2^/df	RMSEA	SRMR	CFI	TLI	PNFI
Standards	<3.00	<0.10	<0.01	>0.95	>0.95	>0.50
Value	1.05	0.03	0.06	0.99	0.99	0.56

**Remarks:** CITC, Corrected Item-total Correlation; C(h²), Communality (h²); SA, Scale Alpha; KMO, Kaiser–Meyer–Olkin; BT, Bartlett’s Test; C%, Cumulative %.

**Remarks:** χ^2^/df, standardized chi-square index; RMSEA, root mean square error of approximation; SRMR, Standardized Root Mean Residual; CFI, comparative fit index; TLI, Tucker-Lewis index; PNFI, parsimonious normative fit index.

Exploratory factor analysis of PPLI Structural Model-5 observed a single factor, KMO 0.89 (>0.6), Bartlett's test of sphericity 0.00 (*p* ≤ 0.05), factorizability of correlation matrix 0.14–0.81 (*r* ≧ 0.13), cumulative total variance explained 72.96% (44, 45).

Validated factor analysis of WHOWBI-5 resulted in a good model fit with factor loading of all items 0.51–1.00 (>0.4), X2/ df 1.05 (<3), RMSEA0.06 (<0.1), PNFI0.56 (≧0.5), CFI0.99 (>0.95), TLI0.99 (>0.95) (44, 45).

The factor loadings of the relevant matrix range from 0.14 to 0.81 (*r* ≥ 0.13), which is below the typical threshold of 0.4 for multidimensional scales; however, the model exhibits good fit. This aligns with the exploratory nature of this study and suggests that the psychological structural characteristics of the audience's perception of PL may be related to HF. To put it more clearly, the audience's PPLI structural model may not be sufficient to support a clear and stable six-dimensional model; however, under the influence of HF, the HFRPAM structural model of the new construct may exhibit a six-dimensional structure.

### 3.2 Risk level

**Figure 5:**
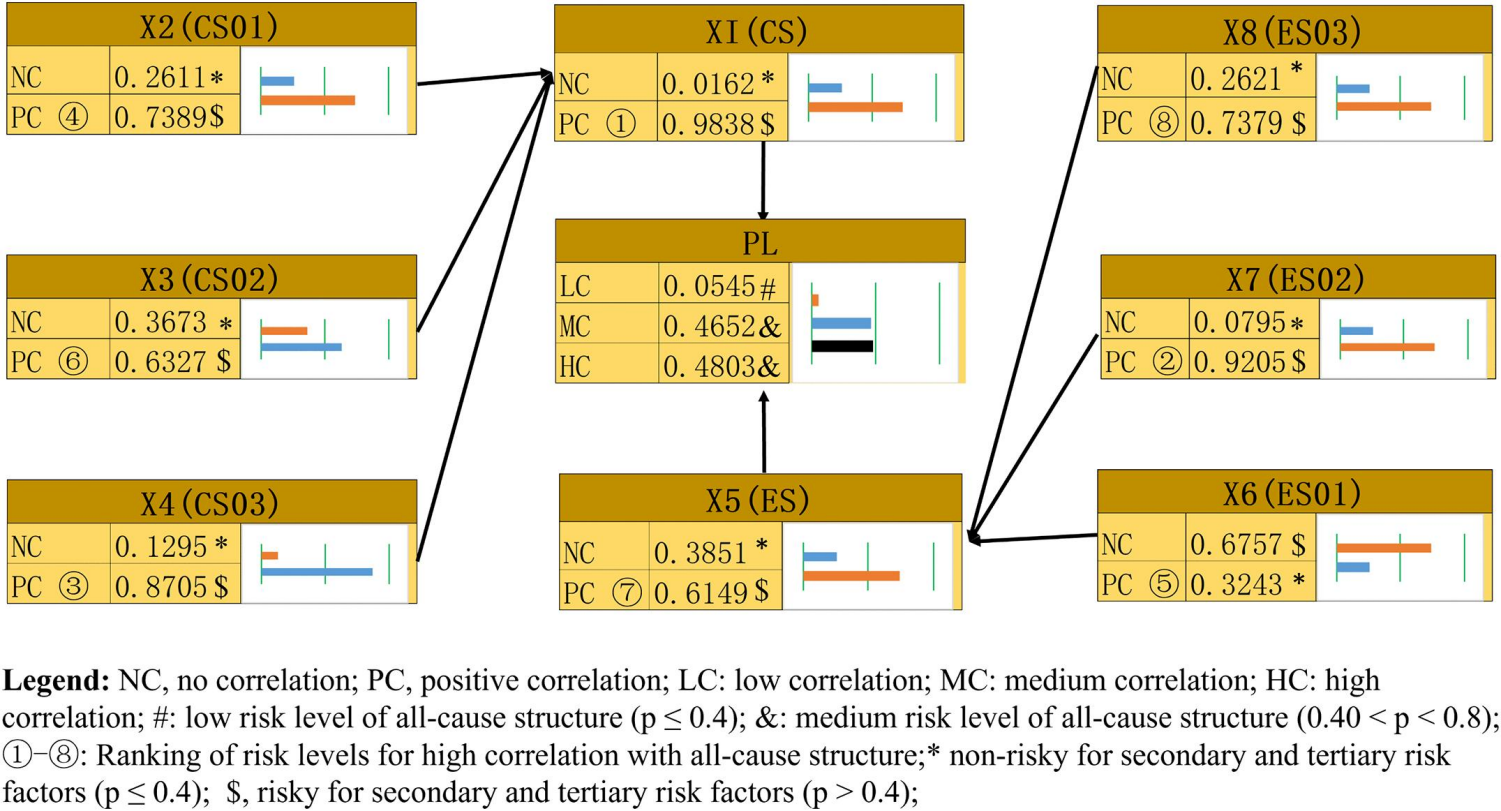
Risk level of human factors risk probabilistic assessment model.

Based on the BNPA calculation results (see Figure 5), the risk level (Research Objective 1) for the all-cause structure of HFRPAM was high correlation (≥4) and medium risk level 0.4803 (0.40 < *p* < 0.8), medium correlation (=3) and medium risk level 0.4652 (0.40 < *p* < 0.8), and low correlation (≤2) and low risk level 0.0545 (*p* ≤ 0.4).

Results of risk levels for the high-correlation (≥4) of the secondary and tertiary indicators in the all-causal structure of HFRPAM: The CS(X1) core stage has a high-risk level of 0.9838 (0.81 ≤ *p* ≤ 1.0), which is the highest; the ES01(X6) sense of self and self-confidence has a low-risk level of 0.3243 (*p* ≤ 0.4), which is the lowest.

### 3.3 Risk weight

Based on entropy reduction Equation 2, the risk weight (Research Objectives 2) of HF in the PL structural model is evaluated in sequence (see Table 4).

**Table 4:** Risk weight (sequence) of human factors using entropy reduction algorithm.

Primary human factors		Secondary human factors		Risk Weight
Structural factors	Encoding	Structural factors	Encoding	Sequence/%
Core stage	X1(CS)			^1^12.82
		Motivation	X2(CS01)	^4^12.48
		Confidence and physical competence	X3(CS02)	^2^12.54
		Interaction with the environment	X4(CS03)	^3^12.52
External stage	X5(ES)			^5^12.46
		Sense of self and self-confidence	X6(ES01)	^5^12.46
		Self-expression and communication with others	X7(ES02)	^5^12.46
		Knowledge and understanding	X8(ES03)	^8^12.25

Remarks: ^1–8^, Sequence/ranking of risk weights for all-cause structures.

This study needs to be honest and say that the result of this risk weight calculation is based on the RP of a single HF.

Under the all-cause structure, the highest risk weight for all HFs was 12.82% for CS(X1) Core Stage, while the lowest was 12.25% for ES03(X8) Knowledge and Understanding.

This result indicates that, under a full-factor framework, there are only minor differences in the risk weights of individual factors. This may be a result of the fact that the audience for this study consists of experts, and the reason may be that it touches upon a key phenomenon in the practical application of the entropy-weighted method: When the observed values of each indicator (the experts' assessments of the HF of PL) show little variation across different samples (i.e., low data dispersion, suggesting that the experts may possess a relatively objective perception of PL), the information entropy approaches its maximum value, resulting in an extremely small “coefficient of variation” and ultimately leading to the calculated weights tending toward the mean

### 3.4 Sensitivity values

Based on the mutual information Equation 3, the HF (Research Objectives 3) of the HFRPAM was evaluated using sensitivity values (see Table 5).

**Table 5:** Using mutual information to calculate human factors activity (sequence).

(A) Sensitivity Value of Synergistic Effects								
Code	X1	X2	X3	X4	X5	X6	X7	X8
X1	NA	0.0249	0.1648	0.1098	0.1098	0.1134	0.0057	0.0249
X2	0.0249	NA	0.2839	0.4798	0.2693	0.1304	0.1098	0.1844
X3	0.1648	0.2839	NA	0.0854	0.3174	0.0936	0.1514	0.2839
X4	0.1098	0.4798	0.0854	NA	0.4501	0.3688	0.2327	0.3652
X5	0.1098	0.2693	0.3174	0.4501	NA	0.3688	0.0964	0.7513
X6	0.1134	0.1304	0.0936	0.3688	0.3688	NA	0.145	0.5567
X7	0.0057	0.1098	0.1514	0.2327	0.0964	0.145	NA	0.1993
X8	0.0249	0.1844	0.2839	0.3652	0.7513	0.5567	0.1993	NA

(B) Human factors Activity (Sequence)								
Code	X1	X2	X3	X4	X5	X6	X7	X8
X1	0/NA	0.0249	0.1648	0.1098	0.1098	0.1134	0.0057	0.0249
X5	0.1098	0.2693	0.3174	0.4501	0/NA	0.3688	0.0964	0.7513
Mean	0.0549	0.1471	0.2411	0.2700	0.0549	0.2411	0.0511	0.3881
**Sequence**	6	5	3	2	6	4	8	1

Based on the synergistic effect of the all-cause structure, the sensitivity values/activity (sequence) resulted in 0.3881 (highest) for ES03(X8) Knowledge and Understanding, 0.2700 (second) for CS03(X4) Interacting with the Environment, 0.2411 (third) for CS02(X3) Confidence and Physical Competence, 0.0511 (lowest) for ES02(X7) Self-Expression and Communication with Others.

The most intuitively expressed HF activity (sequence) is ES03(X8)-CS03(X4)-CS02(X3)-ES01(X6)-CS01(X2)-CS(X1)/ES(X5)-ES02(X7).

A unique and objective phenomenon: ES02(X7) has the lowest sensitivity value but the highest risk level. While this may seem contradictory, it is actually an objective phenomenon: if the ES02(X7) variable itself fluctuates very little, its actual sensitivity value will be low even if it has a significant impact on the outcome; However, if the ES02(X7) variable were to change (e.g., if it were suddenly canceled), the consequences would be extremely severe (high risk of loss), and thus its potential risk level would be high (15).

### 3.5 Optimization Index

Based on the calculation of the sensitivity value, the optimized (comprehensive) index (Research Objectives 4) of HF is further calculated according to the comprehensive index algorithm Equation 4 (see Table 6).

**Table 6:** Use the efficiency-thoroughness tradeoff principle to determine optimal path for human factors.

Index	X1	X2	X3	X4	X5	X6	X7	X8
SV	0.0549	0.1471	0.2411	0.2800	0.0549	0.2411	0.0511	0.3881
RL	0.1282	0.1248	0.1254	0.1252	0.1246	0.1246	0.1246	0.1225
**OR**	0.1831	0.2719	0.3665	0.4051	0.1795	0.3657	0.1757	0.5106
AS	6	5	3	2	7	4	8	1
**OP**								
O → S				2				1
S → O			3			4		
SS	6	5						
OS					7		8	

**Remarks:** SV, sensitive value; RL, risk level; OI, optimization index; AS, activity (sequence); OP, optimization path; SS, Subjective stage; SO, Subjective → Objective; OS, Objective → Subjective; OS, Objective stage.

The results of the HF optimization (composite) index, based on the ETTO of the all-factor structure, were 0.5106 (the highest) for ES03(X8) Knowledge and Understanding, 0.4051 (the second) for CS03(X4) Interacting with the Environment, 0.3665 (the third) for CS02(X3) Confidence and Physical Competence, and 0.1795 (the lowest) for ES02(X7) Self-Expression and Communication with Others (46).

## 4 Analysis

### 4.1 PL and measurement

Although humans have a particularly high fidelity of transmission in the process of cumulative learning, it is not 100% (47). In other words, low sensitivity may result in significant gaps in knowledge transfer, while high sensitivity may pose a high level of risk and require important attention.

Social learning is common in nature, but once a complex culture is established, it can lead to conformity, blind imitation, and excessive credulity (48), thereby triggering safety culture issues. In the risk level assessment of cultural risks, low sensitivity leads to significant gaps in knowledge transfer, while high sensitivity triggers heightened risk concerns; this essentially reflects an imbalance in the system's ability to respond to “change”. If the mutual information is not zero, it indicates that information flow does indeed exist in the teaching process or cognitive transmission—but this is not equivalent to the effective absorption of knowledge or its practical application (i.e., Practical Learning in PL). Currently, there is insufficient evidence to suggest that the “sensitivity” of an individual or system directly determines the effectiveness of knowledge transfer (47, 48). However, in this study, the framework of potential beneficial behaviors (HF-PL-PA, Figure 2), as visualized through the RP of the three HFs—correlation, risk level, and sensitivity value—seems to suggest that HF sensitivity influences the effectiveness of knowledge transfer or PL, or indicates a nonlinear relationship. This includes the occurrence of high correlation with low risk level (the first phenomenon), low correlation with high risk level (the second phenomenon), and the inverse proportional relationship between sensitivity value and risk level/ risk weight.

HFRPAM's high PL correlation (≥4) did not result in a high risk level for HF in the all-cause structure, but had a medium risk level of 0.4803 (0.40 < *p* < 0.8), suggesting that audiences may be at risk for HF during cumulative learning of PL (Research Objectives 1).

This study cannot directly prove safety culture for PL measurement behaviors at this time, but suggests that PL measurements may be worrisome, such as the no correlation of 0.6757 (≤2, no correlation; 0.40 < *p* < 0.8) for ES01(X6) sense of self and self-confidence characterizing a medium risk level. This may suggest that, influenced by HF, PL—which has been studied and promoted for 30 years—could contribute to the achievement of good health and well-being under SDG 3 (6).

The target audience for this study consists of experts, 67.74% of whom (66.74 = 33.87 × 2; Table 2) are policy makers and educators. This finding—where the risk weights under a single framework remain roughly consistent, ranging from 12.25% to 12.82%—may indicate that the concept of PL as a form of quality education/inclusive education has garnered its full/multidimensional attention (3, 4).

As an effective tool for assessing perceptual PL, PPLI also serves as the measurement instrument for the knowledge transfer framework (HF–perceptual PL–PA) in this study. The 6-dimensional, 18-item PL structural model of EFA/CFA presented in this study reflects, first and foremost, the perceptions of PL held by the audience (experts), and secondarily, the indirect effects of HF on PL as an observed variable. The factor loadings in the EFA matrix range from 0.14 to 0.81 (r ≥ 0.13), which is below the typical 0.4 threshold for multidimensional scales; however, the CFA model exhibits good fit, suggesting that the 6-dimensional, 18-item structural model developed by the expert panel may possess relatively comprehensive or beneficial (human-factor) characteristics compared to PE teachers (6), coaches (49), and faculty members (50) regarding the behavioral cognition of perceived PL toward PA.

This study is currently unable to provide direct evidence of the extent to which PL develops/disseminates (measures), or the prevalence of the HF phenomenon in bringing about a lifelong process.

But PL high correlation in three of the eight HFs of HFRPAM characterized by high risk levels, including CS(X1) core stage, ES02(X7) self-expression and communication with others, and CS03(X4) interaction with the environment, may indicate that PL may still have been a complex culture. ES02(X7) self-expression and communication with others has the lowest sensitivity value but the highest risk level. While this may seem contradictory, it is actually an objective phenomenon—a unique yet objective one. To put it another way, if the ES02(X7) variable itself fluctuates very little, its actual sensitivity value will be low even if it has a significant impact on the outcome; however, if the ES02(X7) variable were to change—for example, if attention to HF (such as refraining from communicating with others) were suddenly withdrawn—the consequences could be extremely severe (high probability of loss), indicating a high-risk level (15). This further suggests that Whitehead's assertion that “PL is unmeasurable” may refer more specifically to the complexity of measuring the all-cause structure (24). Therefore, this study recommends conducting further research on measuring the all-cause structure across different demographic groups to elucidate the significance and value of the concept of PL for health and PA.

Bernstein's course and teaching relationship theory argues that assessment/measurement has the function of transmitting valuable knowledge, skills, and understanding (6). The development/dissemination (measurement) of PL is influenced by philosophy, culture, and discourse power (4). ES02 (X7) Self-expression and communication with others 0.0511 (5.11%) sensitivity value, represented the lowest activity (sequence, Research Objectives 3). This seems to indicate that there is still a huge gap in the transfer function of PL as a new term in the PE/education field to facilitate knowledge, skills and understanding. The high-risk level of 0.8705 for CS03 (X4) interaction with the environment seems to further indicate that there is a lack of clarity about whether PL, as an emerging concept, is a help or a hindrance to practitioners. But this study also honestly expresses that the disparity between the risk weights of HFs based on the all-cause structure is not large (Research Objectives 2; highest 12.82, lowest 12.25), which is a useful contribution of PL to an individual's PA, or that the all-cause structure of PL is all given roughly equal weight.

### 4.2 Optimization of PL structural models

PL is an idea based on phenomenal philosophy, has embodied attributes (the body is the object), and claims to have a facilitating function for PA (1). PL is a holistic discourse/culture of PA (4). Whitehead argues that the inner and outer cores of the PL have an interactive function when people engage in PA (1, 42). The research team believes that, as a holistic discourse, the core stage and external stage of PL may have the attributes of subject and object.

Based on the results of the optimization (comprehensive) index, Table 6, the research team discussed the HF optimization path of the PL structural model (Research Objectives 4).

The first HF optimization path, which consists of ES03 (X8) knowledge and understanding and CS03 (X4) interaction with the environment, the active sequence (X8 → X4), has the property of objective-to-subjective change and represents the optimization path in the objective-to-subjective stage. This is similar to the results of the study of the relationship between PL and PE programs (51).

The second HF optimization path, containing CS02(X3) confidence and physical competence and ES01(X6) sense of self and self-confidence, the active sequence (X3 → X6),has the attribute of subjective-to-objective change, and represents the optimization path in the subjective-to-objective stage. This is similar to the results of the correlation study between PL and physical ability in school context (52).

The third HF optimization path, which contains CS01 (X2) motivation and CS (X1) core phases, the active sequence (X2 → X1), has subjective properties and represents the subjective phase optimization path. This is similar to the findings of the full life cycle of traditional sport promotion PL (53).

The fourth HF optimization path, which contains the ES (X5) external nucleus stage and ES02 (X7) self-expression and communication with others, the active sequence (X5 → X7), has objective properties and represents the objective stage optimization path. This is similar to the results of cognitive studies of PL in different words and practices (54).

Building on the optimization indices and optimization paths established in this study, we recommend that future research build upon the cross-sectional observations and explorations presented here to further validate the validity of the chain structure linking HF, PL, and PA using causal models, structural equation modeling, and intervention studies.

### 4.3 Advantages and limitations

Advantages: This study employs quantitative methods to explore the RP characteristics of HF in HFRPAM, including risk levels, risk weights, sensitivity values, and optimization indices, and proposes an optimization approach. PL concepts based on phenomenal philosophy, PA behavior as cumulative human learning, influenced by holistic discourse/culture, philosophical HF. From the perspective of HF and safety culture, this study contributes a beneficial behavioral framework for a comprehensive understanding of PL by employing a chain-like structure linking HF, PL, and PA.

Limitations: Quantitative validation studies conducted using specific population/simple sampling methods (ex-pert-elicitation) have certain limitations. Analyzing HF using a single-factor approach (construct adaptation) limits the ability to consider multiple factors occurring simul-tenuously, resulting in logical limitations. Only the dimensional structure of the HFRPAM was calculated, which is not complete with relatively discrete dependencies. If the HF structure of the items is also calculated and analyzed, combining threshold arbitrariness considerations, the results may be different. This study assumed that its findings were relevant to experts in high-quality PE, including policy experts, education experts, and sports training experts; however, it did not provide direct results from stratified analyses (classified by role) nor from causal models. This constituted a limitation of the study's specific focus. For Bayesian networks based on entropy and mutual information, a sample size of 62 respondents is insufficient; if causal inferences are to be drawn from the network structure of the structural model, the results must be validated with a larger sample.

## 5 Conclusion

The results indicate that, as an exploratory study, the quantitative HFRPAM calculation method proposed in this study can serve as a knowledge translation for current structural models of perceived PA, providing a beneficial behavioral framework to complement the scientific validity of HF in the context of PA. The RP characteristics of the current structural model for PL perception include risk level, risk weight, sensitivity values, and optimization indices, while also offering a strategy for an optimization path to promote PA in PE based on the standard of high-quality education. From the perspective of cultural safety, the framework for potential beneficial behaviors proposed in this study may be the result of recognizing PL as a human factor. By treating HF as a prerequisite or potential element in assessing physical fitness, this approach offers a valuable supplement to safety culture by providing an in-depth analysis of the interactions between motivation and environment underlying physical activities and behaviors. It helps avoid simplistic attributions to “human error” and enables more scientifically sound behavioral assessments. In this study, the results of the risk classification based on the all-cause structure of the HFRPAM may serve as a useful supplement to public health policies implemented by various countries, thereby facilitating the effective promotion of PA. However, using a cross-sectional approach, this study found that a high correlation in the all-cause structure of the HFRPAM did not result in a high-risk level, but rather in a moderate-risk level. This reflects the results of a single audience category (experts) assessing PL at a single point in time; incorporating a wider range of audience types and conducting time-series analyses represent areas for further research.

## Data Availability

The original contributions presented in the study are included in the article/Supplementary Material, further inquiries can be directed to the corresponding author.
